# Prepubertal ultra-low-dose estrogen therapy is associated with healthier lipid profile than conventional estrogen replacement for pubertal induction in adolescent girls with Turner syndrome: preliminary results

**DOI:** 10.1007/s40618-017-0665-3

**Published:** 2017-04-10

**Authors:** Anna Ruszala, Malgorzata Wojcik, Agata Zygmunt-Gorska, Dominika Janus, Joanna Wojtys, Jerzy B. Starzyk

**Affiliations:** 1 Department of Pediatric and Adolescent Endocrinology, Chair of Pediatrics, Institute of Pediatrics, Jagiellonian University, Medical College, Wielicka St. 265, 30-663 Krakow, Poland; 2 Children’s University Hospital in Krakow, Wielicka St. 265, 30-663 Krakow, Poland

**Keywords:** Turner syndrome, Estrogen, Lipid profile, Glucose

## Abstract

**Purpose:**

The metabolic effects of prepubertal low-dose estrogen replacement (LE) therapy in Turner syndrome (TS) have not been fully investigated to date. The present study aimed to compare glucose and lipids metabolism in adolescents with TS on LE and conventional estrogen replacement (CE).

**Methods:**

In 14 TS (mean age 13.8), LE (17β-estradiol, 62.5 μg daily) was introduced before age 12 (mean age 10.5), and followed by a pubertal induction regimen after age 12, and in 14 CE was started after age 12 (mean 14, SD 1.96). Before, and 3 years after starting 17β-estradiol growth velocity, bone age, BMI, and selected parameters of glucose and lipids metabolism were assessed.

**Results:**

There were no significant differences between LE and CE in the mean levels of any parameter before introduction of 17β-estradiol [total cholesterol (TC): 4.1 vs 4.3 mmol/L, LDL cholesterol (LDLc): 2.2 vs 2.4 mmol/L, HDL cholesterol (HDLc): 1.6 vs 1.4 mmol/L, triglycerides: 0.9 vs 1.0 mmol/L, fasting glucose: 4.2 vs 4.4 mmol/L, post-load glucose: 4.8 vs 5.5 mmol/L; fasting insulin: 6.8 vs 8.0 post-load insulin: 21.3 vs 67.0 μIU/mL, HOMA-IR 1.3 vs 1.6]. After three years of treatment, TC and LDLc levels were significantly lower in LE group (3.8 vs 4.4 mmol/L, *p* = 0.004; 1.9 vs 2.4 mmol/L, *p* = 0.03). The other parameters did not differ significantly. There was no negative impact on growth course and bone age advancement nor on BMI in LE group.

**Conclusion:**

Prepubertal LE is associated with healthier lipid profile than CE in girls with TS.

## Introduction

The estrogens are a group of steroid sex hormones that are essential for normal female development. Although their most important physiological role in girls is development of secondary sexual characteristics during puberty, it has been shown that they are secreted by ovaries already in the prepubertal period. The meaning of that phenomenon in healthy girls remains unclear; however, there are some evidence for their contribution in physical and psychological development [1–3]. It is suggested that prolonged estrogen deficiency in girls with hypogonadism that begins in early childhood or even in infancy may have negative effects across many body systems [4]. One of the most common causes of female hypogonadism is Turner syndrome (TS) that occurs in approximately 1/2000 live female births [5]. It results from complete or partial X chromosome monosomy, which in approximately 90% of affected girls causes ovarian dysgenesis and subsequent estrogen deficiency that begins in early infancy [6, 7]. Patients with TS present short stature treatable with growth hormone that makes it possible to reach final height >(−)2.0 SDS in about 62% [8]. Additionally, patients with TS are at risk of metabolic disorders such as glucose intolerance or dyslipidemia [6]. Despite estrogen replacement is a treatment of choice for pubertal induction in adolescent girls with TS since 1960s, many concerns relate to the potential benefits of the initiation of such treatment in younger patients [4, 9]. The studies concerning metabolic effect of the early estrogen replacement are lacking. However, recently published data show that prepubertal low-dose estrogen replacement is more physiologic, and can optimize response to growth hormone treatment, pubertal timing, and improve cognition [4, 10]. Moreover, only early estrogen replacement therapy can prevent osteoporosis and provide proper psychosocial development through slower, more physiological process of puberty [11–16]. Nevertheless, early exposure to the excessive concentrations of sex hormones may have negative impact on growth and pubertal development as well. To date, there are no guidelines supporting the optimal choice of an age of initiation, dosage, or route of administration of estrogen replacement in prepubertal girls with TS. The results of our present study may provide novel insights into metabolic consequences of early estrogen deficiency and its replacement in prepubertal patients with TS.

## Materials and methods

The study conducted in the Department of Pediatric and Adolescent Endocrinology in Children’s University Hospital in Krakow included 28 TS patients [16 with X chromosome monosomy (45,X), 5 with abnormal X chromosome—[three with 46, Xi(Xq), one with 46,XX del (Xq)], and one with 45,X t(3;8)(p23;p11)] and 7 with mosaicism [three with 45,X/46,Xi(Xq) 50/50%, one with 45,X/46,XX 90/10%; 1 with 45,X/46,Xi(Xq) 70/30% one with 45,X/46,X derX 65/35%, and one with 46,XY[27]/45,X[18] 60/40%] treated with human recombinant growth hormone. Before entering the study, in all participants, hypergonadotropic hypogonadism due to ovarian failure was confirmed on the basis of elevated follitropin levels. In the Low Estrogen (LE) Dosage Group (*n* = 14, mean age 13.8, SD 1.55), low dose of oral 17β-estradiol [62.5 μg once daily; Estrofem mite (Novo Nordisk) pills containing 1 mg of 17β-estradiol were splitted by pharmacist] was introduced before the age of 12 (mean 10.5, SD 0.95), and was followed by a standard pubertal induction regimen after the age of 12. In the Conventional Estrogen (CE) Dosage Group (*n* = 14, mean age 16.4, SD 1.64), pubertal induction (Estrofem mite (Novo Nordisk) pills containing 1 mg of 17β-estradiol) was started in accordance with present standards, after the age of 12 (mean 14, SD 1.96).

In all participants before and 3 years after starting 17β-estradiol, total cholesterol (TC), LDL cholesterol (LDLc), HDL cholesterol (HDLc), and triglycerides (TG) were measured in the fasting blood sample by the dry chemistry method with a Vitros 5.1.FF machine (Ortho Clinical Diagnostics, Rochester, NY, USA). LDL cholesterol was measured indirectly.

Standard oral glucose tolerance test was performed with the assessment of fasting (G0) and after 120′ post-load of glucose (G120) and insulin levels (I0, I120). Insulin resistance index (HOMA-IR) was calculated. We also analyzed the course of growth, BMI changes, and bone age advancement during the study period in both groups. The estradiol levels in the blood have not been investigated during the study.

To compare the two sets of data, the *T* student or two-sided Mann–Whitney *U* test was used. The level of significance was set at *p* < 0.05. Calculations were performed using the STATISTICA 12.0 PL software.

## Results

Despite exposure to estrogens in prepubertal age, we observed no negative impact on growth course and bone age advancement nor on BMI in LE group (Table 1). There was no case of premature breast development in LE group. At the moment of 17β-estradiol introduction, the mean height SDS were significantly lower in LE in comparison to CE (−2.2 vs −3.2, *p* = 0.045). One and 3 years after low-dose 17β-estradiol introduction, we revealed no significant impact on growth velocity, contrary to patients receiving standard doses of 17β-estradiol at the age of puberty that presented increase of growth velocity. After 1 year of 17β-estradiol replacement, the advance of the bone age was 1.4 years in LE and 2.4 years in CE; the difference was not statistically significant. After 3 years, the advancement in both groups (LE and CE) was 3.07 and 3.1 years, respectively; the difference was also not statistically significant. There were no significant differences between LE and CE in any assessed parameters before introduction of 17β-estradiol (TC 4.1 vs 4.3 mmol/L, LDLc 2.2 vs 2.4 mmol/L, HDLc 1.6 vs 1.4 mmol/L, TG 0.9 vs 1.0 mmol/L, G0 4.2 vs 4.4 mmol/L, G120 4.8 vs 5.5 mmol/L; I0 6.8 vs 8.0 µIU/mL, I120 21.3 vs 67.0 μIU/mL; HOMA-IR 1.3 vs 1.6). Three years after 17β-estradiol, TC and LDLc levels were significantly lower in LE group (3.8 vs 4.4 mmol/L, *p* = 0.004; 1.9 vs 2.4 mmol/L, *p* = 0.03). The other parameters did not differ significantly (HDLc 1.5 vs 1.6 mmol/L TG 1.2 vs 1.3 mmol/L G0 4.6 vs 4.8 mmol/L G120 5.2 vs 6.0 mmol/L; I0 12.3 vs 15.6 µIU/mL, I120 62.7 vs 83.7 μIU/mL; HOMA-IR 2.5 vs 3.6), for details, see Table 2.

**Table 1:** Comparison of the auxological data in LE and CE groups at the beginning, 1 and 3 years after of different models of 17beta estradiol regimen dosage—low dose in prepubertal and conventional dose in pubertal period

	17βE introduction			1 year after 17βE introduction			3 years after 17βE introduction		
	Mean [SD]		*p*	Mean [SD]		*p*	Mean [SD]		*p*
	LE	CE		LE	CE		LE	CE	
Age [years]	10.6 [0.9]	14 [2.0]	<**0.0001**						
Body height [cm]	129.37 [6.8]	139.3 [9.7]	**0.012**	136.3 [6.5]	144.6 [1.4]	**0.005**	146.8 [9.8]	152.2 [7.2]	0.22
Body height [SDS]	−2.2 [0.9]	−3.2 [1.4]	**0.045**	−2.11 [0.9]	−2.89 [1.0]	0.06	−2.1 [1.1]	−2.04 [1.2]	0.9
Body mass index [kg/m^2^]	17.0 [2.1]	19.3 [5.1]	0.30	18.27 [2.7]	20.23 [5.7]	0.45	20.0 [3.5]	21.8 [5.6]	0.45
Body mass index [SDS]	−0.2 [0.7]	−0.03 [1.5]	0.87	0.07 [0.9]	0.1 [2.0]	0.53	0.18 [1.3]	0.45 [2.1]	0.90
Bone age [years]	10.1 [1.5]	12.1 [1.3]	**0.002**	11.5 [1.2]	13.3 [0.8]	**0.0004**	13.2 [1.7]	14.6 [0.9]	0.8
Age of breast development (ThII)	11.0 [0.6]	13.6 [1.0]	<**0.0001**						

Statistically significant results are bolded

**Table 2:** Comparison of the selected parameters of glucose and lipids metabolism in LE and CE groups at the beginning, and 3 years after of different models of 17beta estradiol regimen dosage—low dose in prepubertal and conventional dose in pubertal period

TC [mmol/L]	17βE introduction			3 years after 17βE introduction		
	Mean [SD]		*p*	Mean [SD]		*p*
	LE	CE		LE	CE	
	4.1 [0.7]	4.3 [0.6]	0.4	3.8 [0.4]	4.4 [0.5]	**0.004**
LDLc [mmol/L]	2.2 [0.8]	2.4 [0.7]	0.6	1.9 [0.4]	2.4 [0.5]	**0.03**
HDLc [mmol/L]	1.6 [0.3]	1.4 [0.3]	0.1	1.5 [0.3]	1.6 [0.5]	0.6
TG [mmol/L]	0.9 [0.4]	1.0 [0.4]	0.4	1.2 [0.5]	1.3 [0.5]	0.8
LDLc/HDLc	1.5 [0.65]	1.8 [0.66]	0.3	1.4 [0.6]	1.7 [1.7]	0.3
G0 [mmol/L]	4.2 [0.5]	4.4 [0.4]	0.2	4.6 [0.4]	4.8 [0.6]	0.3
G120 [mmol/L]	4.8 [1.0]	5.5 [1.2]	0.1	5.2 [1.7]	6.0 [1.7]	0.2
I0 [µIU/mL]	6.8 [1.7]	8.0 [3.0]	0.3	12.3 [5.2]	15.6 [8.5]	0.2
I120 [µIU/mL]	21.3 [15.5]	67.0 [37.2]	0.1	62.7 [52.8]	83.7 [58.8]	0.4
HOMA-IR	1.3 [0.4]	1.6 [0.7]	0.2	2.5 [1.1]	3.6 [2.1]	0.1

Statistically significant results are bolded

Undesirable level (>95th percentile) of lipid fractions in children TC ≥5.17 mmol/L, LDLc ≥3.36 mmol/L, TG ≥1.47 mmol/L, HDL c <0.91 mmol/L [14]

## Discussion

Ovarian dysgenesis is considered as the most important consequence of X chromosome monosomy. The first overt manifestation of the hypogonadism in patients with TS is lack or delay of secondary sexual characteristics development. Nevertheless, estrogen deficiency begins earlier, in early childhood or even in infancy and probably, has many consequences for the general development and well-being. Therefore, there were few attempts of the assessment of the possible effect of the early, before the age of puberty induction of estrogen replacement therapy. Charmian et al. previously demonstrated that individual regimen of low-dose estrogen replacement from the age of 5 caused slower, more physiological tempo of puberty and did not influence the bone age progression, time of menarche, and age of developing breast stages Th > II [4]. Some reports demonstrated the importance of early estrogen replacement for psychosocial development and self-esteem, and better cognitive abilities, e.g., memory, processing speed, and motor performance [15–18]. However, this type of replacement treatment remains an experimental method to date. One of the arguments for the delay of estrogen replacement therapy till the age of puberty is concern about the possible negative impact of early estrogen exposure on skeletal maturation and final growth. That has been denied by Ross et al. in a study that proved favorable impact of prepubertal estrogen replacement combined with growth hormone for the optimization of height gain [10]. Also in our study, we did not observe any negative consequences of prepubertal low-dose estrogen replacement therapy on the growth course. The analysis of the skeletal maturation also showed no deleterious effects of early estrogen introduction. After 1 year of 17β-estradiol replacement, the advance of the bone age in LE group was 1.4 year, less than in CE group (2.4). After 3 years, the advancement in both groups (LE and CE) was comparable: 3.07 and 3.1 years, respectively. Another open question is a role of prepubertal estrogen replacement therapy in prevention of the development of metabolic disorders. This has not been investigated to date. Development of the metabolic disorders is a serious problem in patients with TS as they have a fourfold increased relative risk of the insulin resistance, diabetes mellitus, and subsequent complications, which contribute up to 25% of the causes of death in adult TS patients [19]. Elevated TC and LDLC concentrations in childhood, and especially adolescence, are considered as predicted factors of atherosclerosis in the future [20]. The causes of metabolic disorders in patients with TS tend to be much more complex than in general population. Because of short stature and abnormal body proportions, women with Turner syndrome are more likely to be overweight and obese. However, metabolic disorders such as impaired insulin sensitivity, hypercholesterolemia, and liver steatosis are often diagnosed even in non-obese ones [21, 22]. Some data point that metabolic disturbances may be associated with the origin of the X chromosome monosomy itself. Moreover, patients who retained their maternal X present with higher BMI and lower total and low-density lipoprotein cholesterol [21]. Also insulin resistance is thought to be connected not only with body weight and composition but also with the karyotype [23]. As it was recently shown in survey, overall metabolic risk in normal-weight women with Turner syndrome is lower than in obese peers and some other mechanisms, e.g., FGF 21 may play an important role in this phenomenon [22]. The influence of growth hormone treatment and estrogen replacement on metabolism of patients with TS is significant. Growth hormone through its anabolic activity can improve body proportions, but on the other hand can also increase insulin resistance [21]. However, increase of the insulin growth factor-1 (IGF-1) level, especially within the first months of treatment, may temporary improve insulin sensitivity, even in patients with pre-existing insulin resistance. The impact of estrogen replacement in TS patients on glucose metabolism seems to be more complex and remains unclear. Some authors have shown that estrogen replacement promotes obesity, insulin resistance, and hypercholesterolemia [24]. On the other hand, there are studies negating the relationship between exogenous estrogens and insulin resistance and glucose metabolism disorders [23]. Additionally, recently published metaanalysis of the studies performed in menopausal women has shown that low estrogen replacement has favorable effect on lipid profile in that group of patients [25]. In the present study, we decided to investigate whether low dose (62.5 μg daily) of oral 17β-estradiol replacement would impact the metabolic status of patients with TS. Our results clearly show a beneficial effect of such treatment for lipid profile. There were no significant differences between LE and CE in any assessed parameters of glucose and lipids metabolism before introduction of 17β-estradiol. After three years, mean TC and LDLc levels were still within normal ranges in both groups, however, significantly lower in LE group (3.8 vs 4.4 mmol/L, *p* = 0.004; 1.9 vs 2.4 mmol/L, *p* = 0.03). The main limitations of the present study are relatively small group of participants and lack of serum estradiol levels measurements during replacement therapy. The another issue, that may raise doubts is the real impact of such a small dose of 17β-estradiol for improving the lipid profile. Since the administration of 62.5 ug of 17β-estradiol caused no visible effect on the traditionally considered “estrogen-dependent” tissues as for, e.g., breast tissue or growth cartilage, could it improve the lipid profile? To confirm this observation, further investigation in larger groups of TS patients is needed. Nevertheless, results of the studies that compared low doses of estrogens vs placebo in postmenopausal women show that low-dose estrogen replacement therapy was associated with healthier lipid profile [25]. Despite these limitations, results of the study provide next argument supporting consideration of early estrogen therapy showing no negative impact on growth and skeletal maturation and positive metabolic effects. Further investigations in this field are needed for better understanding of the role of estrogens in the prepubertal period and development of better standards of comprehensive care for patients with Turner syndrome.

## Conclusion

Prepubertal LE is associated with healthier lipid profile than CE in girls with TS. LE should be considered for girls with TS not only due to positive effects on growth and puberty course but also because of its positive metabolic effects.
